# Multistate US Outbreak of Rapidly Growing Mycobacterial Infections Associated with Medical Tourism to the Dominican Republic, 2013–2014^1^

**DOI:** 10.3201/eid2208.151938

**Published:** 2016-08

**Authors:** David Schnabel, Douglas H. Esposito, Joanna Gaines, Alison Ridpath, M. Anita Barry, Katherine A. Feldman, Jocelyn Mullins, Rachel Burns, Nina Ahmad, Edith N. Nyangoma, Duc B. Nguyen, Joseph F. Perz, Heather A. Moulton-Meissner, Bette J. Jensen, Ying Lin, Leah Posivak-Khouly, Nisha Jani, Oliver W. Morgan, Gary W. Brunette, P. Scott Pritchard, Adena H. Greenbaum, Susan M. Rhee, David Blythe, Mark Sotir, the RGM Outbreak Investigation Team

**Affiliations:** Centers for Disease Control and Prevention, Atlanta, Georgia, USA (D. Schnabel, D.H. Esposito, J. Gaines, A. Ridpath, J. Mullins, N. Ahmad, E.N. Nyangoma, D.B. Nguyen, J.F. Perz, H.A. Moulton-Meissner, B.J. Jensen, O.W. Morgan, G.W. Brunette, M. Sotir);; Maryland Department of Health and Mental Hygiene, Baltimore, Maryland, USA (D. Schnabel, K.A. Feldman, D. Blythe);; New York City Department of Health and Mental Hygiene, New York, New York, USA (A. Ridpath, Y. Lin);; Boston Public Health Commission, Boston, Massachusetts, USA (M.A. Barry);; Connecticut Department of Public Health, Hartford, Connecticut, USA (J. Mullins);; Massachusetts Department of Public Health, Boston (R. Burns);; New York State Department of Health, Albany, New York (N. Ahmad);; Montgomery County Health Department, Norristown, Pennsylvania, USA (L. Posivak-Khouly);; Newark Department of Child and Family Well-Being, Newark, New Jersey, USA (N. Jani);; Florida Department of Health, Tallahassee, Florida, USA (P.S. Pritchard);; Johns Hopkins Hospital, Baltimore (A.H. Greenbaum);; Johns Hopkins Bayview Medical Center, Baltimore (S.M. Rhee)

**Keywords:** Mycobacteria, Mycobacterium abscessus complex, Mycobacterium chelonae, Mycobacterium fortuitum, medical tourism, tourist, rapidly growing mycobacteria, nontuberculous mycobacteria, antibacterial drugs, antibiotic, Dominican Republic, United States, cosmetic surgery, healthcare-associated infections, nosocomial infections, bacteria, antimicrobial resistance

## Abstract

During 2013, the Maryland Department of Health and Mental Hygiene in Baltimore, MD, USA, received report of 2 Maryland residents whose surgical sites were infected with rapidly growing mycobacteria after cosmetic procedures at a clinic (clinic A) in the Dominican Republic. A multistate investigation was initiated; a probable case was defined as a surgical site infection unresponsive to therapy in a patient who had undergone cosmetic surgery in the Dominican Republic. We identified 21 case-patients in 6 states who had surgery in 1 of 5 Dominican Republic clinics; 13 (62%) had surgery at clinic A. Isolates from 12 (92%) of those patients were culture-positive for *Mycobacterium abscessus* complex. Of 9 clinic A case-patients with available data, all required therapeutic surgical intervention, 8 (92%) were hospitalized, and 7 (78%) required ≥3 months of antibacterial drug therapy. Healthcare providers should consider infection with rapidly growing mycobacteria in patients who have surgical site infections unresponsive to standard treatment.

## Abstract 2

Infections in 6 states were linked to persons traveling to undergo cosmetic surgical procedures.

Infections with rapidly growing mycobacteria (RGM), which include the species *Mycobacterium* *abscessus*, *M*. *chelonae* and *M*. *fortuitum*, are difficult to diagnose (*1**,**2*) and treat (*3**,**4*). RGMs primarily cause pulmonary or cutaneous infections (*5*). Although symptoms vary and can be nonspecific, the classic cutaneous symptoms include painful nodules that develop into persistent, discharging abscesses (*2**,**4**,**6**,**7*). Systemic symptoms (e.g., fever) are often absent (*2**,**4*). Certain RGMs, including those in the *M. abscessus* complex, are notoriously resistant to most antibacterial drug classes (*5*). Surgical debridement or removal of foreign bodies (e.g., implants) is usually a necessary adjunct to antibacterial therapy (*1**,**4*). Infections are prolonged; median symptom duration is reported as 3–12 months (*4**,**8*).

RGMs, similar to other nontuberculous mycobacteria, are ubiquitous environmental organisms reported worldwide (*5*) and are most frequently detected in nonsterile water sources, including natural waters and engineered water systems (*9*). Infections by these organisms acquired in healthcare settings are most often associated with breeched sterile technique and exposure to nonsterile water (*4**,**10*). Outbreaks in these settings have been reported (*11**,**12*) and include those associated with cosmetic surgeries performed in the United States (*13*) and internationally (*14*). RGM infections acquired by medical tourists, who are persons who travel to another country specifically to receive healthcare (*15*), have been reported (*6**,**16**–**18*). Nevertheless, scope, impact, and character of medical tourism and its public health significance are not well defined (*15**,**19**,**20*).

On August 23, 2013, a physician in Maryland, USA, reported to the Maryland Department of Health and Mental Hygiene *M. abscessus* complex–positive surgical site infections in 2 women who had undergone cosmetic surgery the previous month at a private surgical clinic in the Dominican Republic. These women disclosed that they had an acquaintance in Massachusetts with “similar problems” after a procedure at the same clinic. Concerned that additional unrecognized cases might exist, Department of Health staff consulted with multiple state and local health departments in collaboration with the US Centers for Disease Control and Prevention (CDC) and initiated an investigation. Investigation objectives were to determine outbreak scope of RGM surgical site infections among medical tourists who traveled to the Dominican Republic for procedures, identify epidemiologic links among patients, and mitigate outbreak effect.

## Methods

### Epidemiologic Investigation

This outbreak investigation was determined to be a public health response. Therefore, review by institutional review board was not required. All patients gave informed consent.

After identification of the first 2 patients, measures were taken by the RGM Outbreak Investigation team, which consisted of state and local health departments and the CDC, to locate additional patients who had RGM infections that were associated with cosmetic surgery undergone in the Dominican Republic. Health alerts selective for clinicians, especially those serving Dominican communities, were disseminated through Epi-X (http://www.cdc.gov/epix), a secure notification network for public health professionals; the Emerging Infections Network (http://ein.idsociety.org/), a secure notification network for clinicians; the American Society of Plastic Surgeons (http://www.plasticsurgery.org/); and local public health networks. In addition, health messages encouraging clinicians and patients to report possible RGM infections to local public health authorities were distributed through mainstream and social media outlets. A probable case-patient was defined as a US resident who had a cosmetic surgery procedure in the Dominican Republic during March 2013–February 2014 and a diagnosed soft tissue infection unresponsive to standard antibacterial drug therapy. A confirmed case was defined as a probable case with a culture positive for RGM.

Patients were interviewed verbally by state or local public health authority personnel, who used a standard questionnaire that was designed by the RGM Outbreak Investigation Team to elucidate common exposures or experiences, characterize clinical symptoms and disease courses, and estimate the associated financial burdens. Interviews were conducted in Spanish or English at the patient’s request. A standard medical chart abstraction form was used to review available US medical records to obtain medical histories and document medical and surgical interventions that included antibacterial drugs, clinical encounters, and disease courses. All identified surgical clinics in the Dominican Republic were geolocated by using street addresses to assess for geographic clustering (Google Earth, Mountain View, CA, USA; and ArcGIS, Environmental Systems Research Institute, Redlands, CA, USA). Data from questionnaires and medical chart abstraction forms were entered into a spreadsheet and analyzed. CDC reported findings to the Dominican Republic Ministry of Health (MOH) throughout the investigation. 

### Laboratory Analysis 

Patient wound culture isolates from clinical and public health laboratories were submitted to CDC for organism confirmation and for pulsed-field gel electrophoresis (PFGE) testing. In addition to submitting isolates, the New York City Public Health Laboratory staff analyzed all isolates from New York, NY, USA, by PFGE and sent corresponding PFGE band patterns to CDC for comparison.

**Figure 1:**
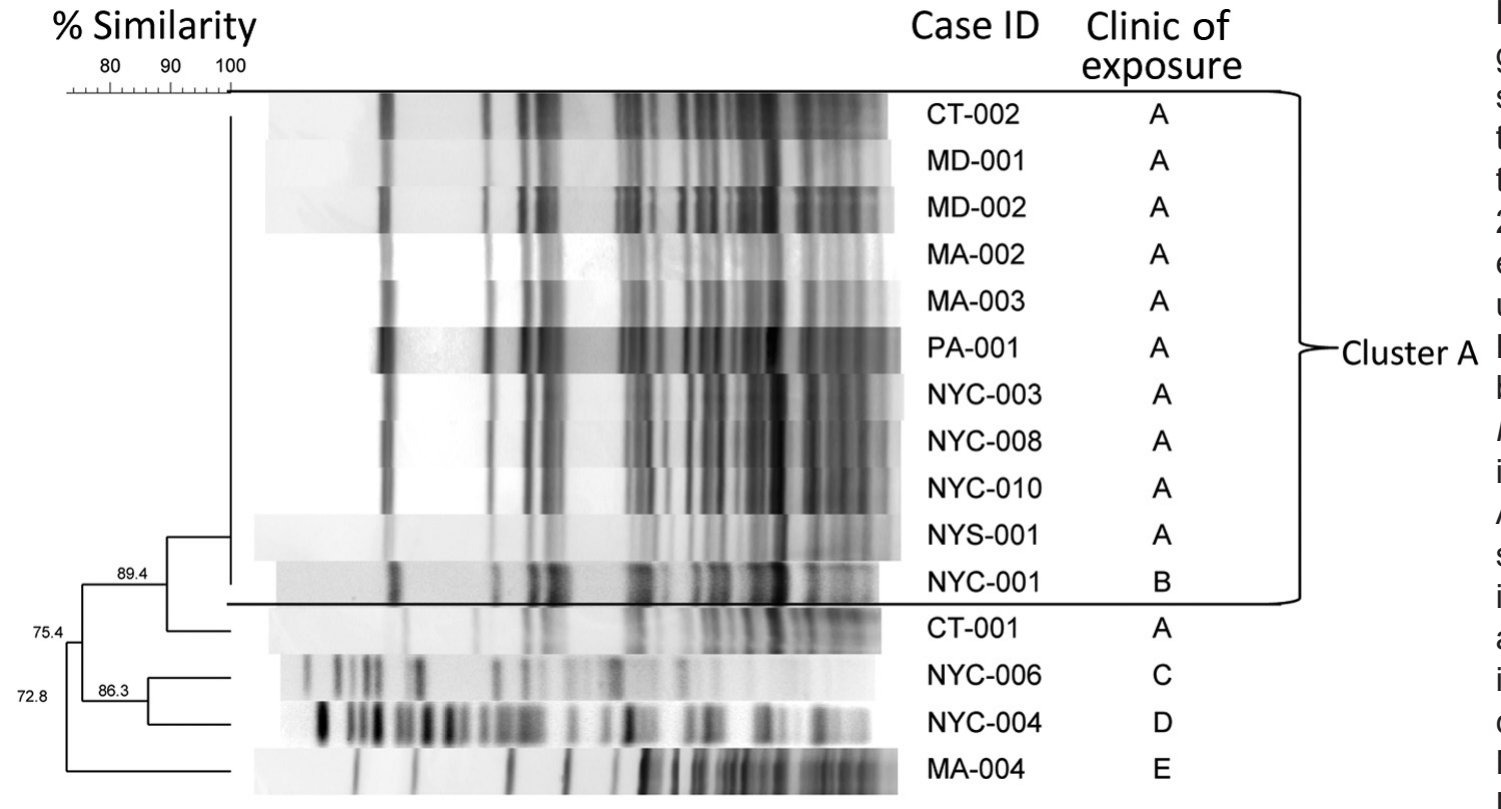
Dendrogram of rapidly growing mycobacteria in surgical site infections among patients in the US associated with medical tourism to the Dominican Republic, 2013–2014. Patients were exposed in 5 known clinics and 1 unknown clinic (data not shown). Pulsed-field gel electrophoresis band patterns for available *Mycobacterium abscessus* complex isolates were restricted with the Asel enzyme and run at 3 and 20 seconds for 20 hours. Isolates with indistinguishable band patterns are labelled cluster A. Case ID indicates US location and patient case number. ID, identification; NYC, New York City; NYS, state of New York.

Isolates were first subcultured onto Middlebrook and Cohn 7H10 Agar (Fisher Scientific, Pittsburgh, PA, USA) and were checked for purity after 7 days of incubation at 30°C. Molecular typing was performed by using PFGE. Molecular chromosomal DNA was prepared as described previously (*21*). Genetic relatedness of the isolates was analyzed by using BioNumerics software (Applied Maths, Austin, TX, USA). PFGE pattern similarity was based on Dice coefficients, and a dendrogram was built by using the unweighted pair group method (Figure 1). The Tenover criteria (*22*) were used to interpret comparison of the patient isolate PFGE patterns; patterns were classified as indistinguishable (100% similarity), closely related (1–3 band difference), possibly related (4–6 band difference), or unrelated (>7 band difference). Use of 16s rRNA and rpoB gene sequencing of representative isolates (on the basis of PFGE patterns) confirmed species of isolates (*23**–**25*).

## Results

### Patient Characteristics

In 6 states, 21 patients (18 confirmed and 3 probable) were identified: New York, 11; Massachusetts, 4; Connecticut, 2; Maryland, 2; New Jersey, 1; and Pennsylvania, 1. Core demographic information was available for all 21 patients, 18 (86%) patients provided questionnaire information, and 3 (14%) declined to be interviewed. Median age of the 21 patients was 40 years (range 18–59 years); all were female (Table 1). Of those for whom data were available (n = 20), all reported US residency for a median of 25 years (range 9–44 years); 15 (75%) patients were born in the Dominican Republic, 2 in the United States, and 1 each in Brazil, Puerto Rico, and Jamaica. Residency and country of origin information were unknown for 1 patient.

**Table 1:** Characteristics of 21 patients in multistate US outbreak of RGM infections after medical tourism to the Dominican Republic, 2013–2014*

Characteristic	Value
Demographics	
Female sex	21 (100)
Median age, y (range)	40 (18–59)
United States resident	20 (95)
Birth country	
Dominican Republic	15 (71)
United States	2 (10)
Other	3 (14)
Missing	1 (5)
US residency,† median y (range)	25 (9–44)

Clinical background, n = 15	
Surgical history	
Previous cosmetic surgeries in Dominican Republic	0 (0)
Any previous cosmetic surgeries	2 (13)
None	8 (53)

Dominican Republic surgical history	
Surgical clinic	
Clinic A	13 (62)
Clinics B, C, D, E	7 (33)
Unknown clinic	1 (5)

Most common procedures‡	
Liposuction	15 (71)
Abdominoplasty	12 (57)
Buttocks augmentation	8 (38)
Breast augmentation	6 (29)
Breast reduction	4 (19)
Median no. procedures received (range)	2 (1–6)

*Values are no. (%) except as indicated. Residency data are missing for 1 patient. RGM, rapidly growing mycobacteria. †Among 15 case-patients whose charts were abstracted. ‡>1 answer possible per patient.

Of the 21 case-patients, 13 (62%) learned of the Dominican Republic clinic where they had surgery through friends or family, 7 (33%) through the Internet, and 1 through a television advertisement. None had previously had cosmetic surgery performed in the Dominican Republic. Of the 16 who reported, cost affected the decision of 15 (94%) to undergo procedures in the Dominican Republic: “a lot” for 9 (56%); “somewhat” for 3 (19%); and “a little” for 1 (6%).

**Figure 2:**
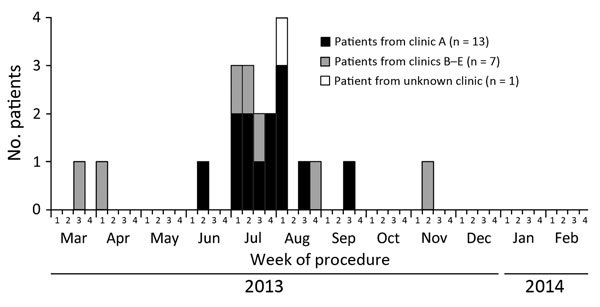
Number of case-patients in the United States who were infected in surgical sites with rapidly growing mycobacteria associated with medical tourism to the Dominican Republic, by procedure week, March 2013–February 2014 (N = 21). Weeks are defined uniformly as week 1, days 1–7 of the month; week 2, days 8–15; week 3, days 16–23; week 4, days 24–28/30/31. Pulsed-field electrophoresis pattern of the *Mycobacterium* *abscessus* isolate from the clinic A case-patient diagnosed during week 2 of June 2003 differed from those of remaining clinic A case-patients who were infected with *M. abscessus*.

Of the 21 case-patients, 13 (62%) underwent surgical procedures at clinic A (Table 1); no common clinic was identified for the remaining 8, although data were missing for 1. No geographic clustering of clinics was observed. All procedures occurred during March 21–November 12, 2013 (Figure 2); 10 (85%) of clinic A patients reported procedures during July and August. Fifteen (71%) case-patients underwent liposuction; less frequent procedures included abdominoplasties, buttocks augmentations, breast augmentations, and breast reduction (Table 1). Eighteen (86%) case-patients had >1 procedure performed.

### Postsurgery and Prediagnosis

All 21 case-patients remained in the Dominican Republic after their surgeries for a median duration of 18 days (range 10–80 days); 14 (67%) stayed with friends or relatives, and the remainder stayed in hotels or guest houses. Of 17 for whom data were available, all had >1 postsurgery follow-up visits at the clinic where their surgery was performed. All but 1 (94%) case-patient reported having a dressing change; 9 of 15 (60%) reported that clinic staff did not wear gloves during a follow-up visit. None of the patients reported observing the use of tap water for wound care or reuse of wound care supplies by clinic staff. For 10 (48%) case-patients who provided their own wound care, none reported using saline, syringes, tap water, or multiuse alcohol while in the Dominican Republic. Seven (33%) case-patients reported bathing in the Dominican Republic; of these, 5 (71%) only sponge-bathed to minimize water exposure to the wounds. All denied swimming in the Dominican Republic. No postsurgical epidemiologic links among patients were described. Patients also were interviewed about their wound care and possible exposures after return to the United States; no common exposures were identified.

### Laboratory Testing

Of the 18 confirmed RGM infections, 16 (89%) were of the *M. abscessus* complex and 2 (11%) were *M. fortuitum*. Surgical site specimens from all 12 (92%) confirmed clinic A patients grew *M. abscessus* complex; specimens from 1 clinic A patient did not grow RGMs, and the patient’s status was classified as a probable case. *M. abscessus* complex isolates from 15 patients were analyzed by using PFGE (Figure 1); 11 (73%) were from clinic A and 4 from clinics B, C, D, and E (Figure 2). Overall, 11 (73%) of 15 isolates had indistinguishable PFGE patterns. Of the 11 clinic A isolates tested, 10 (91%) matched by PFGE. The clinic A patient whose isolate did not match the primary PFGE cluster pattern reported a procedure date 3 weeks earlier than all other clinic A patients (Figure 2). One isolate from a patient whose procedure was not performed at clinic A (NYC-001 in Figure 1) matched the PFGE cluster associated with the clinic A infections.

## Clinical Courses and Treatment

Medical chart abstractions were completed for 9 (69%) of 13 clinic A patients and 1 (12%) of 8 non–clinic A patients; we report data from the 9 available clinic A patients’ charts. Illness onset was a median 24 days (range 1–60 days) after the surgical procedure (Table 2). Among 9 patients for whom we had data, care was sought a median 38 days (range 23–142 days) after the procedure. For 5 of the 9 patients for whom we had data and for whom RGM culture was positive, time to RGM laboratory confirmation was a median of 79 days (range 20–111 days) after the initial US medical encounter.

**Table 2:** Clinical course and therapeutic interventions for patients in multistate US outbreak of RGM infections acquired by medical tourists who underwent procedures in clinic A in the Dominican Republic, 2013–2014*

Characteristics	Value
Time from clinic A surgical procedure	
Median days to illness onset, n = 13	24 (1–60)
Median days to seek care†	38 (23–142)
Median days to RGM diagnosis‡	138 (52–183)

Time course from initial US clinic visit	
Median days to RGM diagnosis§	79 (20–111)

Signs and symptoms,¶ n = 11	
Systemic	
Chills	6 (55)
Malaise	5 (45)
Fever	5 (45)
Localized	
Swelling	10 (91)
Pain	10 (91)
Clear fluid discharge	9 (82)
Scarring	9 (82)
Redness	7 (64)
Warmth	7 (64)
Pus collection	5 (45)

Patient medical history, n = 9#	
No. days hospitalized for RGM infection	8 (92)
1	3 (33)
2–3	3 (33)
>3	2 (22)

No. therapeutic surgical procedures	
1–2	4 (44)
3–5	3 (33)
>5	2 (22)

Types of therapeutic surgical procedures**	
Debridements	6 (67)
Drainage procedures	5 (56)
PICC line	3 (33)
Ultrasound guided aspiration	3 (33)
Implant removal	2 (22)
Abdominal washouts	2 (22)
Missing data	0 (0)

No. antibacterial drug classes used per patient	
<3	0 (0)
4–5	2 (22)
>5	7 (78)

Duration of antibacterial drug therapy, mo	
<1	0 (0)
>1–≤3	2 (22)
>3	5 (56)
Unknown duration	2 (22)

Changed antibacterial drugs	
Yes	7 (78)
No	2 (22)

*Values are no. (%) except as indicated. PICC, peripheral inserted central catheters; RGM, rapidly growing mycobacteria. †10 of 13 (77%) with available data. ‡6 of 13 (46%) with available data. §5 of 13 (38%) with available data. ¶Signs and symptoms reported at a frequency <19% include skin stretching, fluctuance, bleeding from breast (site of surgical procedure), ulcerations, back pain, itching, body aches, and blisters and painful and red nodules that gradually enlarged and dehisced. #Medical charts assessed for only 9 of 13 clinic A patients. **>1 answer possible per patient.

Wound-related signs and symptoms were reported more frequently than systemic signs and symptoms: >80% of case-patients reported swelling, pain, clear fluid drainage, and scarring, but only 45% exhibited systemic symptoms such as chills, malaise, and fever (Table 2). Of 9 (92%) clinic A patients for whom data were available, 8 were hospitalized in the United States; 5 (55%) were hospitalized on >2 occasions (Table 2). All of the 9 underwent >1 therapeutic surgical procedure; 5 (55%) required >3 separate procedures (range 1–11 procedures). Procedures included incision and drainage, debridement, implant removal, ultrasound-guided drainage, and insertions of peripherally inserted central catheters. Of the 9 patients, 7 (78%) required courses of antibacterial drugs >3 months duration, and 7 (78%) were prescribed >5 different classes of antibacterial drugs. A change in antibacterial drug treatment regimens was required for 7 (78%) patients. Of 5 patients’ susceptibility data, all associated infections exhibited resistance or intermediate resistance to most classes of antibacterial drugs tested. Of 13 clinic A patients, 12 (92%) were contacted an average of 9 months after their surgeries (1 patient was lost to follow-up); only 1 (8%) patient reported full recovery when contacted.

### Financial Burden

Financial burdens associated with therapeutic care were examined for 18 (86%) of the 21 confirmed and probable case-patients; 3 (14%) patients did not provide information. Of the 18 who responded, 13 (62%) used medical insurance to pay for treatment of infection in the United States; 3 (14%) paid cash; and 2 (10%) answered “don’t know.” Four (19%) patients reported that their insurer had declined to cover certain costs; 10 (48%) reported that their illness had caused financial problems; and 2 reported that the financial burden was not limited to direct medical costs but that indirect costs (e.g., the inability to work) compounded their financial difficulties.

## Discussion

We identified 21 cases of RGM surgical site infections in 6 US states among medical tourists to the Dominican Republic. Thirteen of the patients underwent procedures at a single clinic, clinic A; most were infected by the same strain of *M. abscessus* complex, potentially from a single, unidentified point source. Most of the procedures at clinic A occurred within a 2-month period. Similar to a previously reported outbreak among “lipotourists,” who had traveled to the Dominican Republic to have liposuction during 2003 (*16**,**17*), the clinic A cluster in this investigation occurred during what might represent a baseline of unrelated cosmetic surgery–associated RGM infections. This baseline might reflect sporadic or systematic failures in hygienic practices at certain surgical centers. 

RGM infection is not a nationally notifiable disease in the United States or Dominican Republic. Therefore, cases described here might represent a limited proportion of those that actually occurred. Cases were dispersed throughout 6 states in the United States and were only identified after active case-finding was initiated, catalyzed by recognition of the initial 2-case cluster by an astute clinician. Health alerts to clinicians and the public associated with the investigation facilitated identification and treatment of some RGM patients. 

This RGM outbreak illustrates potential risks for medical tourists. Little systematically collected data is available about the scope of and risks for medical tourism (*20*). Industry estimates regarding the number of US residents who travel abroad for medical services vary widely, from 75,000 to 750,000/year (*26**,**27*). In 2010, travel of an estimated 4 million medical tourists worldwide/year was reported by the Institute of Medicine, now known as Health and Medicine Division, of the US National Academy of Sciences (Washington, DC, USA) (*28*). Despite this discrepancy in estimated numbers, most reports indicate that the frequency of medical tourist activities and subsequent public health effects will likely increase in the future because of ease of travel, increased marketing and communications, and anticipated cost savings (*15**,**26**,**29**,**30*).

Cost previously has been reported to be the primary driver of medical tourism decisions (*31*) and was the case among our patients: 88% noted that cost affected their decision to undergo surgery in Dominican Republic. Studies have reported a cost savings of ≈28%–88%, depending on destination and services (*32**–**34*). However, among patients in this investigation, cost was not the only factor and possibly not the most important factor. Most patients had friends or family in the Dominican Republic or were originally from the Dominican Republic and learned of the Dominican Republic surgical clinic through word-of-mouth from friends or family; these factors might have played an important role in their decision making.

Both the American Medical Association and American College of Surgeons recommend that prospective medical tourists use internationally accredited facilities (*35*). During our investigation, we were unable to identify any Dominican Republic surgical centers accredited by an internationally recognized accrediting organization. However, standards vary between accrediting organizations, and no published evidence is available that shows improved outcomes are associated with accreditation internationally (*35*). Although accreditation might offer guidance to consumers (*15*), expansion of medical tourism should spur international organizations to understand what aspects of accreditation methods improve patient outcomes and to uniformly apply these across settings. Outbreak activity has also prompted calls to strengthen infection control and safety standards for cosmetic surgery centers in United States (*36*). In addition to enhanced oversight, improved outcome surveillance related to medical tourism and better information about the scope, costs, and safety of the industry are needed to establish guidance for healthcare consumers, payers, healthcare providers, and policy makers (*15**,**19**,**20**,**32*).

The clinical courses of clinic A patients exhibited symptoms, diagnostic delays, and treatment difficulties typical of *M. abscessus* complex infections (*4*). Signs and symptoms among patients were largely cutaneous and localized, but severe in nature, and most case-patients exhibited painful, nonhealing soft-tissue infections; systemic signs and symptoms were less prominent. The 24-day incubation period after surgery for clinic A patients is comparable with that described by previous studies (*10**,**17*). Some patients might have delayed seeking care because of the localized nature and from mild to moderate severity of initial symptoms (*2*). Even after seeking care, some patients experienced a substantial delay in diagnosis. As described in the literature, initial cultures in RGM infections frequently demonstrate no pathogenic organism growth, and clinicians might only consider RGMS after a wound infection fails to respond to typical postsurgical therapeutic interventions (*4*). 

Although RGMs grow well on routine bacterial culture media, clinical specimens frequently fail to exhibit growth after empirical use of common antibacterial drug therapy, particularly when swab specimens are collected instead of body fluids or tissue (*4*). To minimize diagnostic delays, especially when encountering surgical site infections among medical tourists, clinicians should consider RGMs, collect adequate specimens, and communicate this suspicion to ensure correct laboratory testing is performed (*1**,**8*).

RGMs are notoriously antimicrobial drug resistant and difficult to treat (*37**,**38*). The isolates from patients in this investigation were resistant to multiple classes of antibacterial drugs and required protracted and complex antibacterial drug combinations and courses. Surgical interventions are frequently necessary adjuncts to antibacterial drug therapy (*1**,**4**,**39*). Multiple clinic A patients required >2 hospitalizations and multiple surgical procedures. Considering the 6–12-month duration of a typical *M. abscessus* complex disease course (*4*), our finding that only 1 clinic A patient was known to have fully recovered by the close of our investigation was expected.

Because RGMs are ubiquitous environmental contaminants, site inspections to identify inadequate infection control practices (e.g., reuse of equipment or inadequate cleaning and disinfection procedures) and to test water sources are crucial in discovering the point source of an outbreak (*10**,**11*). However, although environmental reservoirs usually serve as a primary source for RGMs, how these organisms are introduced into the patient is often difficult to determine. The tendency of RGMs to cause soft tissue infection in immunocompetent adults after surgical procedures is not understood (*8*). Whereas specific virulence factors among RGMs might predispose the patient to dermal and subdermal infection, such infections could also reflect the propensity of RGMs to form biofilms and relative resistance to disinfectants and surgical antibacterial drug prophylaxis, combined with lapses in infection control (*8**–**11*).

CDC provided epidemiologic information identifying the involved surgical clinics, clinic practices, and patient activities in the Dominican Republic to the Dominican Republic MOH. On the basis of this information, the Dominican Republic MOH performed site visits to certain identified clinics, including clinic A. Although detailed inspection findings were unavailable, and its current status is unknown, the Dominican Republic MOH reported that clinic A was closed after their site visit.

In summary, our investigation identified a cluster of RGMs associated with surgery at clinic A and additional cases associated with other cosmetic surgery clinics in the Dominican Republic. RGM infection remains a potential risk for medical tourism, and clinicians should consider RGMs early, especially among medical tourists. As this investigation demonstrates, treatment of persons with RGM infections is often prolonged and resource-intensive. Patient burdens were not limited to the financial cost of healthcare but also included a loss of ability to work and decreased quality of life during treatment. The extensive number of hospitalizations, drugs, and corrective surgeries required by patients in this study illustrates the considerable burden of illness to individual patients and the healthcare system (*40*). Understanding the role of medical tourism in disease risk and increasing patient protections in this context will require an ongoing effort by the international public health and medical communities. Clinicians and public health officials, particularly those serving communities with connections to immigrants from medical tourism destinations, should be vigilant and consider RGM infections in the differential diagnosis for persons who have wound infections after surgery in these destinations. 
